# Persistence of an Endangered Amphibian in a Fully Anthropogenic Forested Pondscape

**DOI:** 10.1002/ece3.71608

**Published:** 2025-06-27

**Authors:** Tibor Hartel, Cristian Valeriu Maloș, Eliana Sevianu, Ionuț Pascu

**Affiliations:** ^1^ Faculty of Environmental Science and Engineering Babeș‐Bolyai University Cluj‐Napoca Romania; ^2^ Department of Forest Monitoring ‘Marin Dracea’ Romanian National Institute for Research and Development in Forestry Voluntari Romania

**Keywords:** *Bombina variegata*, connectivity, monitoring, occupancy model, patchy population

## Abstract

Anthropogenic habitats can play a pivotal role in species persistence within human‐modified landscapes. We examined aquatic habitat use by *Bombina variegata* , an endangered amphibian that relies entirely on a dense network of 157 human‐made temporary ponds created by historical cart tracks and maintained through ongoing off‐road vehicle activity. Over three consecutive years (2021–2023), including one year of extreme drought (2022), we investigated how pond characteristics and connectivity influenced pond occupancy, reproduction, and colonization–extinction dynamics in this anthropogenic pondscape. Pond availability closely followed precipitation: wet years (2021, 2023) supported ~25 ponds/km^2^, while drought in 2022 led to a 90% reduction in pond formation. Adult occupancy was high in 2021 and 2023 (*ψ* > 0.8), and egg/larval occupancy in 2021 reached *ψ* = 0.70. Detection probabilities exceeded 0.75 for adults and 0.5 for larvae. In 2021, adult occupancy was best explained by connectivity metrics, suggesting dispersal‐driven pond use. By 2023, occupancy was influenced by both connectivity and pond‐specific features (hydroperiod, area, depth), indicating a shift toward selective site use. Between 2021 and 2023, the colonization rate was 0.50 and the extinction rate was 0.13. These findings confirm *B. variegata* 's flexible breeding strategy and highlights the ecological value of anthropogenic pond networks for patchy populations. Conservation efforts should focus on preserving both the spatial configuration (connectivity) and functional properties (hydroperiod, pond density) of such systems. A key challenge is to manage human disturbance to sustain pond formation while avoiding stressing the amphibians and habitat degradation.

## Graphical abstract

We studied *Bombina variegata* in a deciduous forest landscape with dense anthropogenic temporary ponds (~25 ponds/km^2^) over three years. Pond occupancy and reproduction were influenced by pond characteristics, connectivity, and precipitation‐driven pond density. Connectivity drove adult occupancy in 2021, while pond‐specific traits and pond connectivity shaped adult pond occupancy in 2023. Egg and larval presence was driven by pond‐specific variables (2021). Conservation should focus on maintaining pond density, hydroperiod, and connectivity to enhance resilience against climatic extremes.

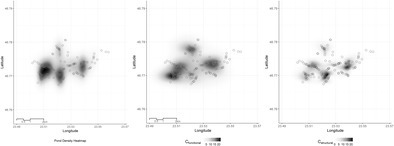

## 1 Introduction

Temporary ponds are highly dynamic environments, characterized by alternating wet and dry periods. Life‐history theory predicts that organisms adapt to such habitat unpredictability through a combination of physiological flexibility, reproductive strategies, and dispersal mechanisms (Stearns 1992; Bohonak and Jenkins 2003; West‐Eberhard 2003; Brendonck and De Meester 2003; Forsman 2015). For example, bet‐hedging strategies may evolve to minimize reproductive failure across fluctuating conditions, while reaction norms for environmental tolerance allow individuals to adjust their phenotypic traits in response to environmental variability (Stearns 1976; Gabriel and Lynch 1992; Barandun and Reyer 1997).

Metapopulation theory explains population persistence as occurring through networks of local habitat patches connected by dispersal, which can stabilize (or even rescue from extinction) local populations (Levins 1969; Hanski 1998; Ovaskainen and Hanski 2001). In classic metapopulation models, local extinctions are balanced by recolonization from neighboring patches, ensuring regional persistence despite local extinctions (Ovaskainen and Hanski 2001). However, many population systems do not conform to a strict metapopulation framework. Patchy population systems are formed by several interconnected habitats where dispersal between patches is intense, even in the short term (i.e., within a season) (Petranka and Holbrook 2006; Smith and Green 2005). In such systems, breeding groups are demographically interconnected to the extent that they function as a single large population rather than as independent subpopulations undergoing local extinction and recolonization dynamics (Harrison 1991). Local extinction events therefore can be quickly balanced by high dispersal rates, and individuals frequently shift between habitats in response to environmental changes or disturbances (Petranka et al. 2004; Petranka and Holbrook 2006).

Pond‐breeding amphibians serve as important model organisms for understanding population dynamics in unpredictable environments. They have complex life cycles, typically comprising three stages: egg, larval (aquatic), and adult (terrestrial) (Wilbur 1980). Their physiology introduces unique constraints, such as permeable skin that functions as both a respiratory and osmoregulatory organ. This characteristic makes them highly sensitive to desiccation, pollutants, and environmental variations. For amphibians breeding in temporary ponds, the pond hydroperiod is a critical factor affecting reproductive success. It not only determines habitat availability but also shapes larval environmental conditions, directly influencing metamorphosis and the fitness of terrestrial life stages (Scott 1994; Van Buskirk 2011). Terrestrial habitats are also vital, serving as feeding grounds, refuges against extreme climatic conditions, overwintering sites, and dispersal corridors (Smith and Green 2005; Marsh and Trenham 2001; Griffiths et al. 2010).

Amphibians are frequently studied within a patchy population framework, where local (i.e., pond) populations undergo cycles of extinction and recolonization (Sjögren‐Gulve 1994; Smith and Green 2005; Marsh and Trenham 2001). However, some studies suggest that the classic metapopulation model (Hanski 1998) may not fully capture the dynamics of many amphibian populations. Certain species display high dispersal rates and therefore exhibit patchy population structure and dynamics (Petranka and Holbrook 2006; Pellet et al. 2007). In patchy population systems, variables reflecting pond quality (local habitat) as well as the influence of the surrounding habitats on habitat use (structural and functional connectivity) should be considered to understand the habitat occupancy and spatiotemporal dynamics of amphibian populations (Pope et al. 2000; Moor et al. 2024).

This research presents a three‐year study conducted in a dense network of temporary ponds to examine aquatic habitat use by the temporary pond breeder *Bombina variegata* . The study system is characterized by four key features: (i) Proximity to a large city: the study system is located at cca 3 km from a major city. (ii) Complete reliance on human‐created temporary ponds: the species relies exclusively on anthropogenic ponds, historically formed and maintained through horse‐cart activity, and more recently by (intensifying) off‐road vehicle activity. Natural disturbance‐sustained ponds are absent. (iii) Climatic variability: the study area exhibits increasing climatic variability, including precipitation extremes in key periods for amphibian life history. This trend can potentially have strong implications for the persistence of the temporary ponds and the studied population system. (iv) Conservation paradox: the focal species is internationally protected and classified as endangered, yet its persistence in this system is tied to a form of human disturbance (off‐road vehicle activity) typically viewed as environmentally harmful from a conservation standpoint.

The overarching objective of this study is to evaluate how pond‐specific characteristics and landscape connectivity influence pond occupancy, reproduction, and colonization–extinction dynamics of *B. variegata* across three consecutive years in a fully anthropogenic pond network. By examining temporal patterns before and after an extreme drought year, we identify a shift from connectivity‐driven habitat use to increased importance of pond‐specific features, reflecting a flexible breeding strategy.

## 2 Materials and Methods

### 2.1 The Model Species

*Bombina variegata* primarily inhabits temporary ponds formed by natural or anthropogenic disturbances, such as logging activities, off‐road vehicle tracks, or historical practices like horse‐cart movements (Hartel and von Wehrden 2013; Cayuela et al. 2018; Barandun and Reyer 1997). These ponds are typically shallow, sunny, and prone to drying, conditions that limit predator presence and create favorable breeding sites (Hartel et al. 2007; Barandun and Reyer 1997). In landscapes where natural disturbance regimes have declined, *B. variegata* increasingly relies on anthropogenic disturbances that sustain breeding ponds (Cayuela et al. 2018). This reliance exposes the species to habitat loss risks when anthropogenic activities cease or when various activities destroy breeding habitats (Cayuela et al. 2018). Additionally, intensive vehicle traffic, while contributing to the formation of breeding sites, can negatively affect body condition, morphology, and stress physiology (Cayuela et al. 2017). The species demonstrates flexibility in habitat use; breeding frequently occurs in ponds with predictable hydroperiods, but ephemeral ponds are also utilized opportunistically when conditions are favorable (Hartel 2008; Barandun and Reyer 1997). The long‐term viability of local (patchy) populations depends on networks of accessible and interconnected breeding sites (Moor et al. 2024; Barandun and Reyer 1997). *B. variegata* is a short‐distance disperser, typically moving 100–200 m among ephemeral ponds (Hartel 2008; Cayuela, Arsovski, et al. 2016a; Cayuela, Boualit, et al. 2016b). In a Romanian forest landscape, Hartel (2008) reported mean between‐pond movements of cca 161 m (172 m for females), varying from cca 93 m to 251 m depending on rainfall and pond availability, with maximum distances exceeding 1 km. Beshkov and Jameson (1980) found within‐season movements averaging cca 64 m for males and cca 20 m for females, increasing to cca 200–300 m post‐breeding, and exceeding 1 km over multiple years. Jacob et al. (2009) documented annual dispersal of up to 732 m for males and 438 m for females, with median movements of cca 79–86 m. The developmental period from spawning to metamorphosis in *B. variegata* is influenced by environmental conditions, particularly temperature and water availability. The number of days until metamorphosis reported in field studies was 33 to 58 days (Barandun and Reyer 1997), with the months of metamorphosis being June and July. This closely aligns with the 30–60 days reported by Hartel (2008) in an experimental study. Higher larval densities result in longer developmental times, and the presence of older tadpoles negatively influences the growth and development of younger ones, delaying metamorphosis and increasing mortality risks (Hartel 2008).

### 2.2 Study Area

**FIGURE 1:**
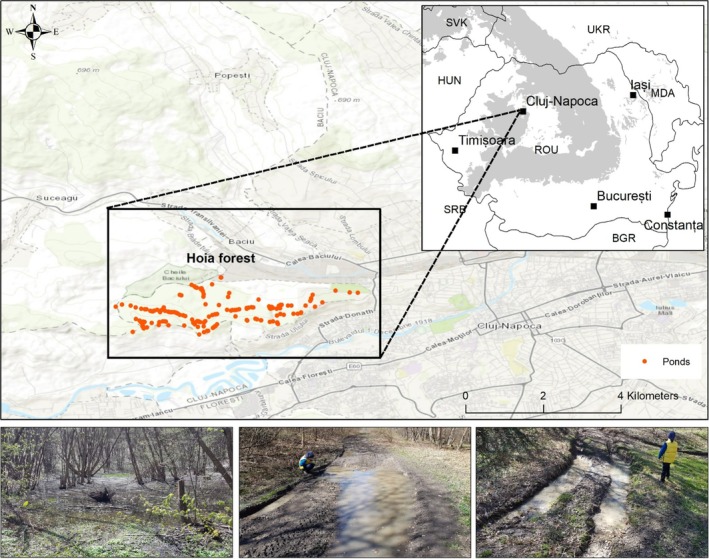
The map of the study area, within the general context of Romania and Europe. Dots on the map Hoia forest are the ponds.

**FIGURE 2:**
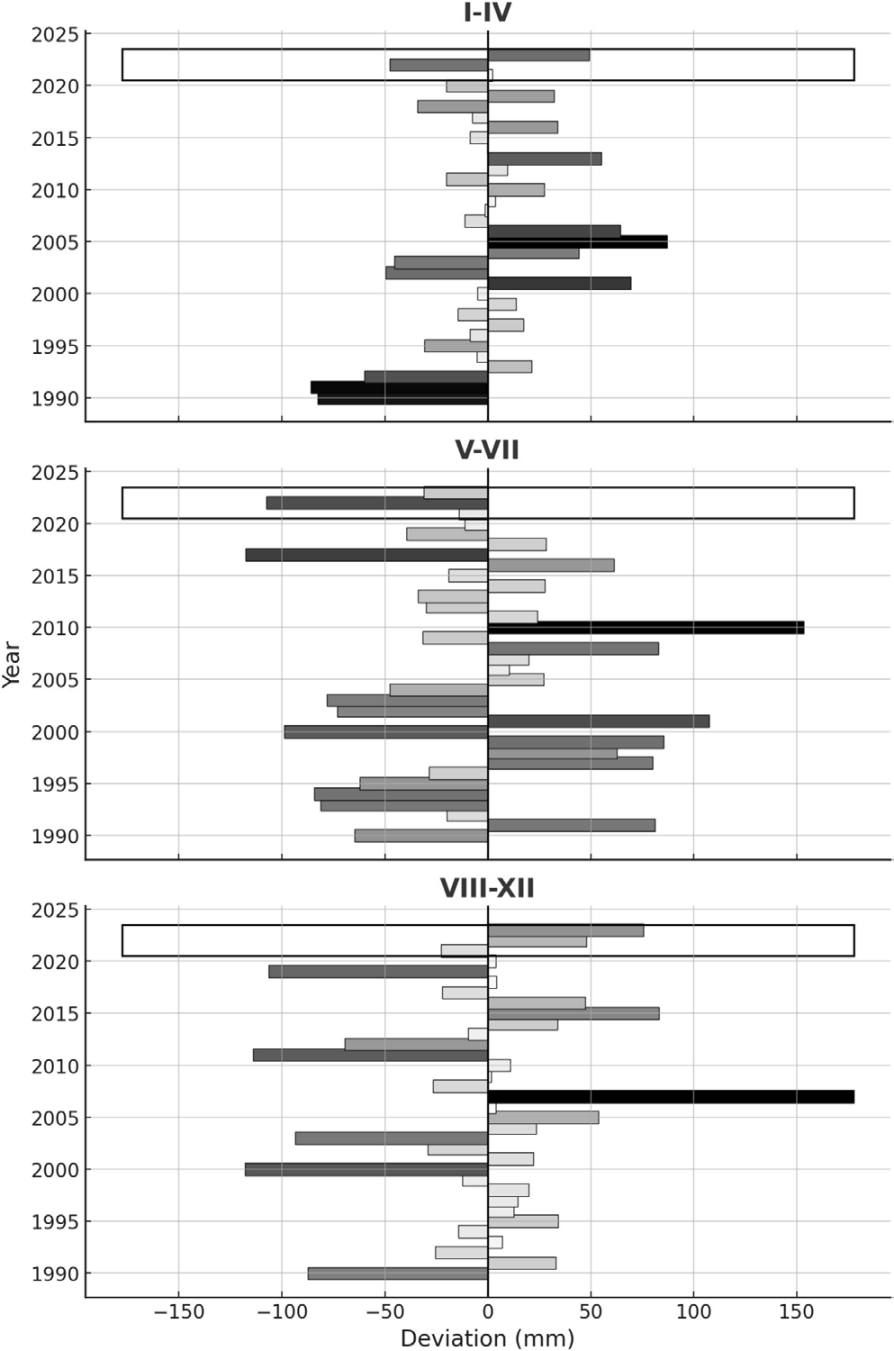
Seasonal precipitation deviations from baseline (1990–2023). Three periods are distinguished from the perspective of the studied toad: January–April (period of spring pond filling), May–July (egg deposition and larval period), and August–December (summer and winter precipitations). The 2021–2023 period is highlighted.

The study area is approximately 6.3 km^2^, covering the Hoia forest in the northwestern part of the city of Cluj‐Napoca (over 300,000 inhabitants) (Figure 1). The elevation of the study area ranges from 372 to 540 m. The area experiences a temperate climate influenced by western air masses, with an average annual temperature of 8.6°C, peaking at 19.1°C in July and dropping to −3.5°C in January. Annual precipitation averages 540 mm, with peaks in June and July and lower levels in March and October. Under normal climatic conditions, snow cover lasts approximately 55 days annually (Micle et al. 2020). However, during our study period (2021–2023) the snow cover lasted less than 10 days every year. The amounts of precipitation for the period of 1990–2023, highlighting three periods of the year relevant to the study organism (i.e., months January–April important for pond filling, months May–July, important for reproduction and metamorphosis, and months July–December, important for soil humidity, and terrestrial environment quality) are presented in Figure 2. The north‐facing slopes of the forest are dominated by oak‐hornbeam forests ( *Quercus petraea* , *Carpinus betulus* ), while the south‐facing slopes support Pannonian oak forests with *Quercus pubescens*. Historical management practices, such as pollarding and coppicing, have shaped the forest's structure, contributing to its biocultural heritage. Additionally, calcareous grasslands hosting rare plant species are interspersed on the southern slopes. These diverse habitats, shaped by both natural and anthropogenic influences, contribute to the forest's ecological and cultural significance.

### 2.3 Surveys

The study was conducted over three consecutive years, from 2021 to 2023. Preliminary surveys began in February each year, to assess the potential for pond formation (Gollmann et al. 2000). Formal amphibian surveys were carried out every year from early April until August 1st, coinciding with the breeding and metamorphosis periods of *B. variegata* , a species known to breed during spring and early summer (Barandun and Reyer 1997). The primary method used was visual encounter surveys, which allowed for efficient observation of all life stages (eggs, larvae, metamorphs, and adults) given the small size of most ponds. All ponds in our study were small (on average < 8 m^2^; see Table 1), allowing full visual inspection from the margins without entering the water. During each survey, observers approached the pond slowly and conducted a thorough scan of the water surface, edges, and surrounding vegetation. Adults were generally easy to detect due to their movement and high visibility in shallow, open ponds (average depth < 20 cm, Table 1). Eggs and larvae were searched for along pond margins and on submerged vegetation. When surface turbidity or canopy reflections limited visibility, dip nets were used selectively, primarily in larger or shaded ponds. Survey effort was sufficient to support high detection probabilities (see Results), and detection histories were used to estimate occupancy per pond per year. The dataset includes both naïve detection (based on observed presence) and model‐estimated occupancy, which adjusts for imperfect detection (see Section 2.5.1).

**TABLE 1:** Descriptive statistics of pond habitat variables and connectivity metrics in 2021 and 2023. Mean, standard deviation (SD), and coefficient of variation (CV) are shown.

Year	2021 (*n* = 157)			2023 (*n* = 157)		
Statistic	Mean	SD	CV	Mean	SD	CV
Area (m^2^)	7.05	8.73	1.23	7.40	8.80	1.18
Maximum depth (cm)	18.87	8.71	0.46	18.91	8.85	0.46
Hydroperiod (days)	82.00	13.03	0.15	81.97	13.04	0.15
Elevation (m)	458.19 (SD = 39.20, CV = 0.08)					
Nearest‐neighbor distance (m)	53.08	55.09	1.03	53.08	55.09	1.03
Functional connectivity	14.94	4.73	0.31	14.55	4.69	0.32
Structural connectivity	10.00 (SD = 4.74, CV = 0.47)					

In total, 157 temporary ponds were surveyed during the study. In 2021, variable field capacity meant that not all ponds received the same number of visits: most ponds were visited 3 to 5 times during the breeding season, but some had fewer visits. In contrast, in 2023, a structured survey design was implemented where all 157 ponds were visited exactly three times. Multiple daylight site visits are rutinously employed to account for imperfect detections in *B. variegata* (e.g., Cayuela et al. 2018). The timing of surveys in both years was aligned with *B. variegata* 's known breeding phenology, from early April through early August. Visits were adjusted to occur approximately 3–8 days after precipitation events to maximize detection probability, as adult breeding activity and pond occupancy are highly rainfall‐dependent in this system (ponds are entirely rainfall‐dependent) (Hartel et al. 2007). This timing aligns with previous observations that identified breeding migrations and the filling of temporary ponds as being closely linked to rainfall patterns, which are essential for successful reproduction (Cayuela, Arsovski, et al. 2016a; Cayuela, Boualit, et al. 2016b).

Metamorphosis was recorded as a binary outcome (1 = observed, 0 = not observed) for each pond. Metamorphs were identified visually along pond margins and in adjacent terrestrial zones. The presence of metamorphs was not inferred from larval occupancy alone but was directly confirmed through the identification of metamorphs. The percentage of ponds with successful metamorphosis was calculated relative to those that had supported larvae earlier in the season.

### 2.4 Variables

#### 2.4.1 Pond‐Related Variables

We recorded key pond variables in the field, such as the area, maximum depth, and hydroperiod (weeks) (Table 1). Pond‐related variables were measured in the field during 2021, 2022, and 2023. Pond surface area was estimated visually in the field during the time of peak hydroperiod, typically mid‐to‐late April–the first 10 days of May. The area was estimated in square meters by visually projecting a reference quadrate over the pond. This method was straightforward since most of the ponds indeed had very small surface areas (formed along dirty roads, created by vehicles). The larger ponds (i.e., over 20 × 20 m) were measured with GPS‐based perimeter mapping. Maximum pond depth was measured at the deepest visible location using a graduated pole. Hydroperiod (in weeks) was defined as the total duration during which standing water was present at each pond, based on repeated visits at a minimum weekly and maximum biweekly intervals. In 2021 we estimated the hydroperiod of each of the 157 ponds. In 2023 a subset of 60 core ponds was visited weekly to increase the accuracy of hydroperiod estimates. A pond was considered dry once no surface water remained. If desiccation occurred during larval development, the presence of deceased tadpoles was recorded, providing evidence of failed metamorphosis.

Given that both 2021 and 2023 were characterized by above‐average precipitation (conditions that favor prolonged pond formation; see Figure 2), and that direct field experience and field photographs confirmed that ponds were maximally filled, we re‐measured area, maximum depth, and hydroperiod for subset of 60 ponds in the eastern half of the study area during the 2023 breeding season. For the remaining ponds, we retained 2021 measurements, as hydrological conditions appeared comparable in both years. Between‐year paired t‐tests showed no significant change in maximum depth (*p* = 0.86) or hydroperiod (*p* = 0.79). Surface area increased modestly yet significantly (9.50 m^2^ in 2023 vs. 8.63 m^2^ in 2021, *p* = 0.04). Within each year (2021, 2023), the 60 ponds that were re‐measured were, on average, larger than the remaining ponds (*p* < 0.05) because the largest ponds were included in the re‐measured sample. However, they did not differ in depth or hydroperiod (*p* > 0.8). Consequently, we infer that the key habitat attributes influencing toad occupancy (pond size in terms of depth and duration) did not differ meaningfully between 2021 and 2023 despite the small increase in surface area. We considered that from an ecological perspective, 2023 and 2021 were not different regarding the measured pond variables.

S*ource ponds* were defined as those that retained water throughout the breeding and larval development period, from early April to early August, thus ensuring sufficient hydroperiod for metamorphosis to occur. 15 such ponds were identified across the study area, the majority of which never dried during any of the three study years. In 2022, when the drought resulted in the premature disappearance of every pond, only these 15 ponds retained water, and each of them produced metamorphs. For the period of the study, 2022 was the year when the source character of these ponds could be apparent, since in this year cca 90% of the temporary ponds dried due to drought.

Besides the variables used for this study (Table 1) we also recorded the presence‐absence of aquatic predatory insect larvae (most notably *Odonata* and *Dytiscus*). However, as their presence was restricted mainly to the 15 source ponds and did not affect the aquatic habitat use (adult site occupancy, metamorphosis, also confirming two previous studies in similar systems, Hartel et al. 2007), we skipped this variable from further analysis.

#### 2.4.2 Connectivity Variables

We used three connectivity metrics to explore the broader population network influence on local pond occupancy of adults and larvae: the nearest–neighbor distance, a structural and a functional connectivity.

The *nearest‐neighbor distance (m)* was defined as the Euclidean distance to the geographically closest neighboring pond. *Structural connectivity* (*C*_structural_). In accordance with the framework of Zamberletti et al. (2018), we quantified structural connectivity using a binary graph‐theoretical approach. Each pond was treated as a node in a pond network, and links (edges) were established between ponds that were within 250 m of each other, a threshold distance that corresponds to the approximate annual dispersal capability of *B. variegata* (Hartel 2008; Jacob et al. 2009). This 250 m threshold captures ecologically relevant movement potential over short timescales (e.g., within‐season or interannual colonization), making it a robust estimate for short‐term metapopulation connectivity (see model species description). For each pond *i*, we calculated this structural connectivity, defined as the number of other ponds located within 250 m.

$$C_{\text{structural},i}=\sum_{j\neq i}1\left(d_{\mathrm{ij}}\leq 250\;m\right)$$

where 1(·) is the indicator function and *d*_*ij*_ is the Euclidean distance between ponds *i* and *j*. This metric reflects the position of a pond within the network and the number of potential sources of colonists, without incorporating occupancy data.

*Functional connectivity* (*C*_functional_). For the purposes of functional connectivity modeling, occupancy was defined as the observed use of a pond by adult *B. variegata* during a given year, based on multi‐visit detection histories. Occupancy status was determined separately for each year (2021 and 2023, as these years were modeled) and is not assumed to persist across years. A pond was marked as occupied if at least one detection was recorded during the study season. Recognizing that both occupied ponds and source ponds (those consistently producing metamorphs across multiple years) contribute to the persistence of *B. variegata* populations, we employed a functional connectivity index (*C*_functional_) that incorporated both components. Specifically, we allowed ponds classified as “occupied” each year, as well as the 15 identified source ponds, to contribute to the connectivity value of each focal pond. Therefore, while accounting for all occupied ponds, we assigned a greater weight (*ws*, see below) to source ponds, reflecting their assumed disproportionate role in ensuring recolonization potential and metapopulation stability. This weighting approach aligns with established concepts in metapopulation theory, where patches contributing to population persistence over time have elevated significance (Moilanen and Nieminen 2002; Pellet et al. 2007). The recognition that connectivity extends beyond spatial proximity to include functional contributions, like reproductive success and consistent occupancy, has been emphasized in recent ecological connectivity frameworks (Keeley et al. 2021; Ovaskainen and Hanski 2001).

$$C_{\text{functional},i}=\sum_{j\neq i}\exp\left(-\alpha d_{i,j}\right)∙\left(O_{j}+w_{s}S_{j}\right)$$

where *d*_*i,j*_ is the Euclidean distance between ponds *i* and *j*, *α* = 1/dispersal distance (250 m), is the distance decay parameter, controlling how quickly connectivity decreases as distance increases. This parameterization follows established dispersal modeling frameworks, where a higher *α* results in steeper distance decay (Moilanen and Nieminen 2002; Ovaskainen and Hanski 2001), *O*_*j*_ is an occupancy indicator with a value of 1 if the *j*‐th pond is occupied and 0 if not. *S*_*j*_ is a source pond indicator, set to 1 if the pond produced metamorphs consistently across three consecutive years, following empirical observations of population persistence and reproductive success, *ws* is a weighting factor that reflects the greater contribution of source ponds to population connectivity. We set *ws* = 3 for those ponds that had breeding success over three consecutive years, if these have a disproportionately higher long‐term influence on the persistence of the whole population system.

Summary statistics of the pond variables for 2021 and 2023, when the pond availability for amphibians was maximum, are presented in Table 1.

### 2.5 Statistical Analysis and Interpretations

#### 2.5.1 Occupancy Models

To evaluate the influence of temporary pond characteristics and connectivity (see Table 1) on pond occupancy (*ψ*), we employed single‐season occupancy models (MacKenzie et al. 2002). All continuous covariates were standardized to improve model convergence and comparability. Collinearity between variables was assessed using Pearson correlation coefficients, and no significant collinearity was detected (all |*r*| < 0.7, the commonly suggested threshold for collinearity concerns, Dormann et al. 2013). Therefore, no variable was removed for statistical analysis.

We built several candidate models (Table 2) using pond, elevation, and connectivity variables (Table 1) to explore factors influencing *B. variegata* occupancy. Separate models were created for adults (2021, 2023) and eggs and larvae (2021). We initially fitted a null model, assuming constant occupancy and *p* across all ponds, to establish a baseline for comparison. Subsequently, single‐variable models were constructed to assess the individual effects of each habitat covariate on pond occupancy (Table 1). Next, we developed more complex models, incorporating habitat and connectivity variables (Table 2). Model selection was performed using the Akaike Information Criterion corrected for small sample sizes (AICc), with models having ΔAICc ≤ 2 considered competitive. The best‐supported models were further examined for ecological relevance.

**TABLE 2:** Model selection results for pond environment and connectivity variables affecting the pond occupancy of *B. variegata* (2021 and 2023).

Model	K	AICc	ΔAICc	AICcWt	Cum.Wt	LL
Adult 2021						
*ψ*(*C*_functional_)	3	534.47	0.00	1.00	0.81	−264.15
*ψ*(*C*_structural_)	3	539.38	4.91	0.08	0.06	−266.61
*ψ*(Nearest_neighbor_distance)	3	540.37	5.89	0.05	0.04	−267.10
*ψ*(Hydroperiod)	3	541.61	7.14	0.03	0.02	−267.73
*ψ*(Null model)	2	542.05	7.57	0.02	0.02	−268.98
*ψ*(Elevation)	3	542.79	8.32	0.02	0.01	−268.32
*ψ*(Max_depth)	3	543.35	8.87	0.008	0.01	−268.59
*ψ*(Full model)	9	545.47	10.00	0.004	0.003	−263.12
*ψ*(Area)	3	544.05	9.80	0.01	0.003	−268.95
*ψ*(Area, max_depth, hydroperiod)	5	545.73	11.25	0.003	0.002	−267.66
*ψ*(Area, max_depth, hydroper, elevation)	6	546.94	12.47	0.001	0.001	−267.19
Eggs and larvae 2021						
*ψ*(Area, mfuna_xdepth, hydroperiod)	5	617.36	0.00	1	0.57	−303.48
*ψ*(Max_depth)	3	619.37	2.02	0.36	0.20	−306.61
*ψ*(Area, max_depth, hydroperiod, elevation)	6	619.45	2.09	0.35	0.20	−303.44
*Ψ*(Full model)	9	624.95	7.56	0.22	0.01	−302.86
*ψ*(Hydroperiod)	3	635.64	18.28	0.00	0.000	−314.74
*ψ*(Area)	3	644.00	26.64	0.00	0.00	−318.92
*ψ*(Null model)	2	647.55	30.19	0.00	0.00	−321.73
*ψ*(*C*_functional_)	3	647.73	30.37	0.00	0.00	−320.79
*ψ*(Nearest_neighbor_distance)	3	647.79	30.43	0.00	0.00	−320.82
*ψ*(*C*_structural_)	3	649.50	32.15	0.00	0.00	−321.67
*ψ*(Elevation)	3	649.54	32.18	0.00	0.00	−321.69
Adult 2023						
*ψ*(Full model)	9	506.97	0.00	1.00	0.92	−243.87
*ψ*(Hydroperiod)	3	513.47	6.50	0.03	0.03	−253.20
*ψ*(Area, max_depth, hydroperiod, elevation)	6	514.96	7.98	0.01	0.01	−251.20
*ψ*(*C*_functional_)	3	516.12	9.14	0.01	0.00	−254.98
*ψ*(Area, max_depth, hydroperiod)	5	516.86	9.88	0.00	0.00	−253.23
*ψ*(Elevation)	3	518.62	11.64	0.00	0.00	−256.23
*ψ*(Max_depth)	3	520.38	13.40	0.00	0.00	−257.11
*ψ*(Null model)	2	521.96	14.98	0.00	0.00	−258.94
*ψ*(Nearest_neighbor_distance)	3	523.44	16.47	0.00	0.00	−258.64
*ψ*(Area)	3	523.53	16.56	0.00	0.00	−258.68
*ψ*(*C*_structural_)	3	524.02	17.05	0.00	0.00	−258.93

Although we conducted repeated surveys and recorded detection histories, we did not collect survey‐specific covariates (e.g., temperature, rainfall, time of day) needed to model variation in detection probability (*p*). To minimize detection bias, surveys were conducted during periods of peak *B. variegata* activity, typically 3–7 days after rainfall events and, during drought conditions, in the late afternoon (after 18:00), when toads were more likely to be active near remaining water bodies. This standardized approach likely reduced major fluctuations in detection probability across ponds and survey periods. For additional discussion of detection limitations, see the Section 6.

For the year 2021 we modeled the pond occupancy by adults as well as the pond occupancy of eggs and larvae (taken altogether). For 2023 we modeled only the adult presence in the ponds because the presence of eggs and larvae was not constantly assessed.

We did not include 2022 in the occupancy modeling due to both statistical and ecological limitations. Statistically, only 15 ponds were available, all of which were occupied and produced metamorphs. This complete lack of variation (100% occupancy) makes modeling inappropriate, as standard occupancy models rely on variation in site use to estimate the influence of covariates (see above). Ecologically, the species used every available pond in 2022 (see below), likely as a survival response to habitat scarcity during extreme drought. As such, no selective habitat use could be inferred that year, and the pattern is best interpreted descriptively rather than modeled.

We estimated the minimum number of surveys required to confidently declare a site as unoccupied, using the formula:

$$N_{\min}=\frac{\log\left(1-0.95\right)}{\log\left(1-p\right)}$$

where *N*_*min*_ represents the minimum number of surveys required to confidently declare a site as unoccupied, 0.95 is the chosen confidence level (95%), and *p* is the detection probability (Reed 1996).

#### 2.5.2 Finding the Hydroperiod‐Threshold for Metamorphosis

To identify the minimum pond duration required for successful metamorphosis, we estimated a hydroperiod threshold using logistic regression. Specifically, we calculated the number of weeks (starting from early April) at which the predicted probability of detecting metamorphs first exceeded 50%. To assess the robustness of this estimate, we applied a bootstrap procedure with 1000 resamples, yielding a median threshold with a 95% confidence interval. For visualization purposes, we fitted a standard logistic model and overlaid the threshold and confidence bounds. While we recognize that this approach simplifies complex developmental dynamics, such as density‐dependent growth and interannual variation in larval development, it nonetheless provides a practical, field‐based estimate of the minimum hydroperiod needed to support successful metamorphosis in *B. variegata* .

#### 2.5.3 Colonization and Extinction Events (2021 and 2023)

To assess temporal dynamics, we quantified colonization and extinction rates between 2021 and 2023. 2022 was skipped because most of the ponds were physically unavailable for toads due to drought (see above). The colonization rate (*c*) and extinction rate (*e*) were calculated to assess temporal changes in site occupancy between 2021 and 2023.

Colonization rate (*c*) represents the proportion of sites that were unoccupied in 2021 but became occupied in 2023 (0 → 1):

$$c=\frac{\text{Newly occupied sites}\;\left(2023\right)}{\text{Total unoccupied sites}\;\left(2021\right)}$$

Where new occupied sites are ponds that were empty in 2021 (0) but became occupied in 2023 (1).

Extinction rate (*e*) represents the proportion of sites that were occupied in 2021 but became unoccupied in 2023 (1 → 0):

$$e=\frac{\text{Lost occupied sites}\;\left(2023\right)}{\text{Total occupied sites}\;\left(2021\right)}$$

where lost occupied sites are ponds that were occupied in 2021 (1) but became empty in 2023 (0).

All statistical analyses were performed in R (R Core Team 2024).

## 3 Results

### 3.1 Pond Density and Precipitation: A Descriptive Presentation

**FIGURE 3:**
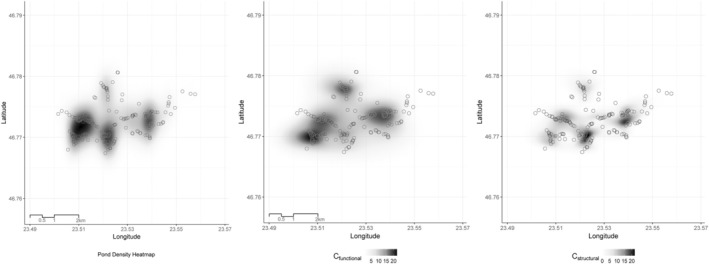
Maps of the study area: Pond density heatmap (A), the ponds according to their *C*_functional_ (B), and ponds according to their *C*_structural_. 2021 is shown as an illustration.

In the study area (6.26 km^2^) the pond density in the years when every pond was formed (2021, 2023) is *cca* 25 ponds/km^2^. The density of occupied ponds (i.e., at least adults were identified in them) in 2021 was *cca* 21/km^2^ and *cca* 20/km^2^ in 2023, while the density of source ponds (2021, 2022, 2023) was *cca* 3/km^2^. A visual inspection suggests a high degree of interconnectedness of these ponds across the study area (Figure 3). The variation of temporary pond density coincides with the relative amount of precipitation in the key periods of pond formation and persistence for the life cycle of the toad (Figure 2). Precipitation from January to April, essential for pond formation, was above the normal average in 2021 (slightly above, Figure 2) and 2023 (much above, Figure 2), matching the higher densities of ponds observed (see above). In contrast, the extreme drought characterizing 2022, the period between May and July (Figure 2) coincides with very low pond densities for this critical period for eggs and larval development (see above).

### 3.2 Adult Pond Occupancy (2021, 2022, 2023)

For adults, the *naïve* occupancy for 2021 was 0.85, while for 2023 it was 0.81. In 2022, only 15 ponds were formed. All were occupied and supported successful reproduction and metamorphosis. We excluded 2022 from modeling due to the very small sample size and the lack of variation in occupancy and reproductive outcome (see above, the modeling part of the Methods).

The model‐estimated occupancy (*ψ*) was high in both 2021 and 2023, with values of 0.89 (SE = 0.03) and 0.82 (SE = 0.03), respectively. Similarly, the detection probability (*p*) is high for both years: 0.78 (SE = 0.02) in 2021 and 0.80 (SE = 0.02) in 2023. Applying the detection probability estimates to the survey effort calculations, we found that slightly fewer than two surveys per site (1.97 in 2021 and 1.86 in 2023) were required to reach 95% confidence in determining species absence. Since most sites (140) in 2021 and all sites in 2023 were surveyed at least twice during the optimal activity period for *B. variegata* , we concluded that our survey effort met the necessary requirements for reliable absence inference for adults.

In 2021, adult *B. variegata* pond occupancy was primarily driven by functional connectivity (Table 2), which had a positive influence on occupancy probability (Table 3). By 2023, the full model, which included both pond variables as well as the connectivity variables, received the strongest support (Table 2). Within this model, structural connectivity and elevation had positive effects on occupancy, while nearest‐neighbor distance had a negative effect (Table 3). Hydroperiod also had a marginally positive influence, suggesting that pond persistence contributed to pond use in 2023. Although not all predictors in the full model were individually significant (Table 3), their combined contribution improved the model's explanatory power.

**TABLE 3:** Parameter estimates for amphibian presence models across years and life stages (2021 and 2023).

Model	β	SE	*Z*	*p*
Adult 2021				
Intercept	2.39	0.41	5.80	< 0.001
*C*_functional_	0.83	0.29	2.86	0.004
Eggs and larvae 2021				
Intercept	1.74	0.40	4.28	< 0.000 1
Area	0.67	0.45	1.49	0.1327
Max_depth	1.59	0.45	3.46	0.0005
Hydroperiod	0.41	0.25	1.60	0.11
Adult 2023				
Intercept	2.12	0.41	5.12	< 0.0001
Area	0.30	0.39	0.76	0.44
Max_depth	0.31	0.34	0.91	0.35
Hydroperiod	0.49	0.27	1.82	0.07
Elevation	0.67	0.34	1.94	0.05
*C*_functional_	1.07	0.26	0.40	0.68
*C*_structural_	1.31	0.43	3.05	0.002
Nearest_neighbor_distance	−1.97	0.44	−2.67	0.007

### 3.3 Eggs and Larvae Occurrence (2021)

The naïve occupancy for eggs and larvae was 0.68, while *ψ* was 0.70 (SE = 0.04) and *p* was 0.57 (SE = 0.03). Based on this detection probability, four surveys would be required to infer the absence of eggs and larvae from the ponds with 95% confidence. However, in 2021 only 15 ponds received four surveys, suggesting that the absence may have been underestimated in ponds with fewer visits. The egg and larval occurrence in ponds was primarily influenced by the model containing pond area, hydroperiod, and pond depth (Table 2). Notably the maximum depth (Max_depth) had a significant positive effect on egg and larval occurrence (Table 3).

### 3.4 Hydroperiod and Metamorphosis (2021)

**FIGURE 4:**
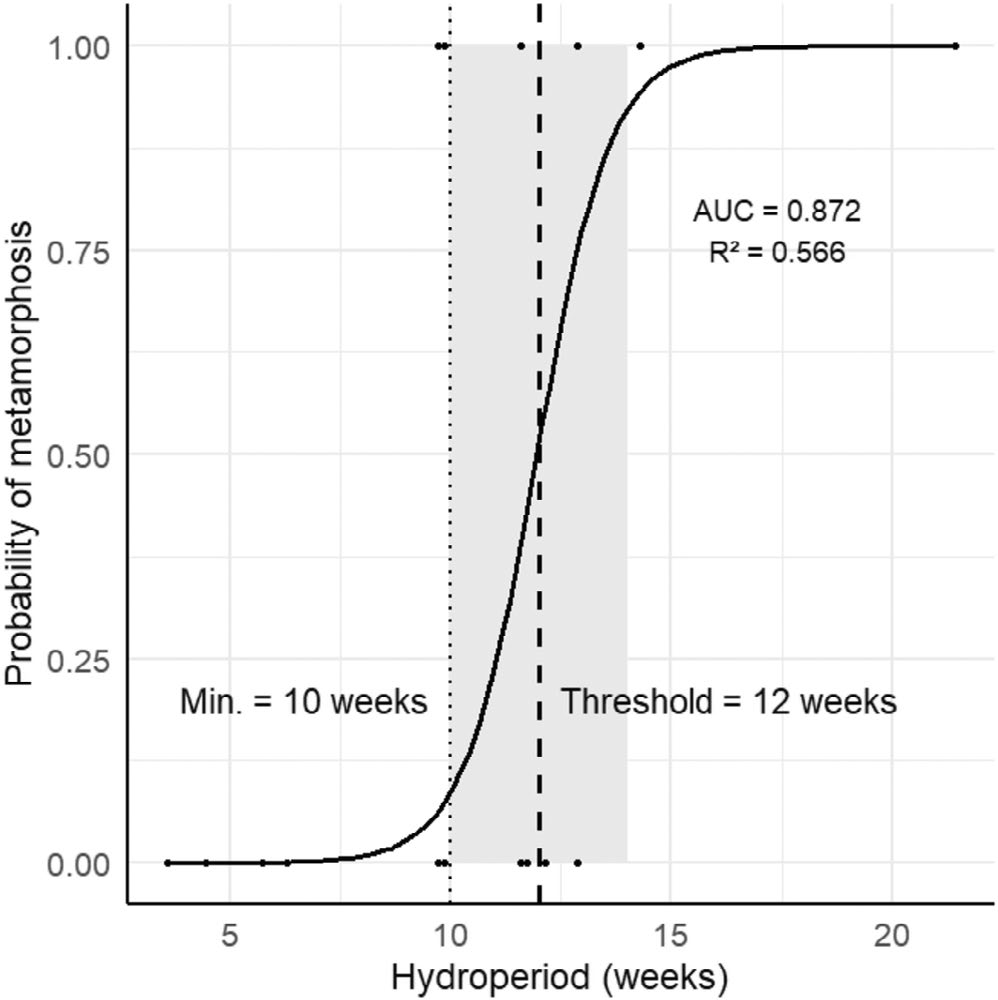
The relationship between hydroperiod and the probability of metamorphosis. *R* ^2^ represents the Nagelkerke *R* ^2^, indicating model fit. AUC represents the discriminatory power of the model.

Metamorphosis occurred in 43.95% of the studied ponds. We found a median threshold of cca 11.71 weeks (95% CI = 9.5–13.5) after which the probability of metamorphosis sharply increases (Figure 4).

### 3.5 Colonization and Extinction (2021 and 2023)

Based on the records of adults, a total of 12 colonization and 18 extinction events were recorded in the years 2021 and 2023. The colonization rate was found to be 0.5 (50% of the unoccupied ponds in 2021 were occupied in 2023) while the extinction rate was 0.13 (13.5% of the occupied ponds in 2021 became unoccupied in 2023). We found no statistically significant difference between the habitat and connectivity features of the ponds with colonization and extinctions (Kruskal‐Wallis ANOVA test, *p* > 0.05 for every variable, data not shown). During the study period, 5 new temporary ponds were formed (within 150 m distance from existing ponds), and the colonization of the new ponds (measured as the presence of adult toads) happened one year after their appearance.

## 4 Discussion

### 4.1 High Pond Density Supports Viable Amphibian Populations

The high density of ponds observed in our study (~25 ponds/km^2^ in 2021 and 2023) provides a potentially favorable pondscape structure for *B. variegata* , especially given its limited dispersal ability and the generally suitable terrestrial habitat in the surrounding forest (see Methods). Although pond density was not included as a direct covariate in our models, the spatial configuration observed (numerous closely spaced ponds with average inter‐pond distances < 250 m) is consistent with conditions known to support metapopulation viability. Moor et al. (2024) showed that amphibian colonization success and persistence are significantly enhanced when ponds are within 0.5 km of at least two other occupied sites, increasing colonization probability by ~3.5 times compared to more isolated ponds. Our data also showed strong interannual variation in pond availability that coincided with precipitation extremes. Years with above‐average precipitation (2021, 2023) supported high pond density, while the extreme drought in 2022 resulted in a drastic reduction in breeding sites. Although precipitation and drought were not modeled explicitly, this temporal change suggests a potential role of climate variability in shaping breeding habitat dynamics. This aligns with previous findings that extreme droughts during key reproductive periods can reduce recruitment and increase extinction risk in pond‐breeding amphibians (Griffiths 1998; Crawford et al. 2022). The consistently low density of source ponds (~3/km^2^) observed across years may reflect such climatic constraints and point to a vulnerability under future drought scenarios. While our findings do not directly measure population resilience or long‐term demographic outcomes, the spatial distribution of source ponds (those supporting metamorphosis across multiple years) emphasizes the importance of well‐connected and hydroperiod‐stable breeding sites. These source ponds likely function as demographic anchors that facilitate recolonization and sustain genetic exchange across the network (Moor et al. 2024). Maintaining both high pond density and functional connectivity will be critical for enhancing the persistence of *B. variegata* in increasingly variable climatic conditions.

Although 2022 was excluded from modeling due to its statistical limitations (*n* = 15, all ponds occupied), it plays a crucial role in understanding the recoverability of this population after a short‐term drought. In 2022 an extreme drought eliminated cca 90% of temporary ponds, yet all remaining ponds (i.e., source ponds) were fully occupied and produced metamorphs. Importantly, occupancy rebounded to > 80% in 2023 when pond density increased again. This pattern, based on the variables we measured, suggests that *B. variegata* can persist through short‐term habitat collapse by concentrating breeding in key refugial ponds. The ability to maintain high occupancy across such short term fluctuating conditions reflects some resistance to interannual environmental variability. However, we acknowledge that longer term effects such as delayed genetic consequences, may not be apparent witihn the temporal extent of our study. Thus, while 2022 could not be modeled statistically, its qualitative contribution supports, but does not definitely confirm our interpretation that the population functions as a highly connected patchy system with potential for rapid functional recovery.

### 4.2 Flexibility in Breeding Pond Use

Our results highlight the flexibility of *B. variegata* in pond use. In 2021, a year characterized by high precipitation, adult pond occupancy was primarily influenced by connectivity metrics, reflecting the importance of dispersal across the available pond network. This pattern suggests that during periods of high pond availability, *B. variegata* exhibits a colonizer strategy, dispersing broadly and utilizing multiple ponds (Barandun and Reyer 1997; Hartel 2008; Cayuela et al. 2018). Amphibians in patchy populations tend to exploit newly available habitats, especially when connectivity facilitates movement across a network of wetlands (Petranka and Holbrook 2006). In such systems, high dispersal capacity is a key trait supporting population persistence (Sinsch 2014). In 2023, however, following the extreme drought of 2022, pond occupancy remained high, but besides the connectivity, it was also driven by pond‐specific characteristics such as hydroperiod, area, and depth. Such behavior reflects increased selection for stable ponds with reliable hydroperiods (Gollmann et al. 2000; Hartel 2008; Petranka et al. 2007; Cayuela, Arsovski, et al. 2016a; Cayuela, Boualit, et al. 2016b). Compensatory recruitment mechanisms (Cayuela et al. 2022) suggest that populations recovering from disturbances increase breeding efforts in more predictable habitats, enhancing persistence. Petranka et al. (2007) also noted that following drought‐induced recruitment failures, amphibians increase breeding efforts in ponds with established hydroperiods to mitigate future risks. This behavior likely explains why the full model (i.e., the one containing pond‐related as well as connectivity variables) was the best in explaining pond use in 2023 (Brooks et al. 2023).

### 4.3 The Occurrence of Eggs and Larvae

The occurrence of *B. variegata* eggs and larvae in ponds during 2021 was best explained by a model that included pond‐specific variables (hydroperiod, area, and depth). This result highlights the importance of stable habitats for reproductive success through larval survival. The significance of pond depth and hydroperiod in breeding site selection aligns with findings from Hartel et al. (2011), who emphasized that longer hydroperiods and greater pond depth enhance larval survival rates. Similarly, Barandun and Reyer (1997) showed that *B. variegata* selects breeding ponds with hydroperiods aligning with larval development timelines, optimizing metamorphosis success. Our finding that the median hydroperiod for metamorphosis was 11 weeks (with a minimum of 9 weeks) is consistent with developmental durations observed in previous studies (Barandun and Reyer 1997; Gollmann et al. 2000).

### 4.4 Colonization and Extinction

The observed colonization and extinction dynamics highlight the species' capacity to navigate a heterogeneous and temporally dynamic pond network. Despite the relatively close average pond distance (211 m) within the considered dispersal threshold of 250 m, colonization and extinction events were notable, indicating that dispersal alone does not guarantee pond occupancy. Moilanen and Nieminen (2002) show that connectivity is necessary but not sufficient for colonization, as habitat quality and stochastic events also influence outcomes. The relatively high colonization rate (0.5) and low extinction rate (0.13) suggest a resilient population, capable of rapidly exploiting new habitat opportunities (Cayuela et al. 2018). The rapid colonization of newly formed ponds in the study area, occurring within a year of their appearance (*personal observation*), further supports this view. Such swift responses to habitat opportunities reflect an adaptive advantage in unpredictable environments (Barandun and Reyer 1997, Petranka et al. 2007). The lack of statistically significant differences in habitat and connectivity features between colonized and extinct ponds suggests that other factors, such as microhabitat conditions or random demographic events, may shape these dynamics (Pellet et al. 2007; Brooks et al. 2023).

## 5 Implications of a Conservation Paradox

Our findings highlight that the long‐term viability of *B. variegata* populations in anthropogenic pondscapes depends on maintaining both high pond density and strong inter‐pond connectivity. Conservation planning must therefore address both pond‐scale habitat attributes (e.g., hydroperiod, depth, and substrate) and landscape‐scale connectivity to promote functional metapopulation dynamics, as emphasized by Petranka and Holbrook (2006). While ephemeral ponds support reproduction in wet years, more stable ponds with extended hydroperiods act as refuges and sources during droughts (as shown by Hartel et al. 2011, for a different system). Maintaining a heterogeneous mix of pond types is essential for buffering interannual climatic variability.

A key conservation paradox emerges in such anthropogenic systems: the very disturbances, such as off‐road vehicle activity, that create and maintain these temporary ponds may also negatively affect toad populations. Cayuela et al. (2017) showed that intensive vehicle traffic significantly reduces *B. variegata* body size and condition and alters endocrine stress responses, with potential consequences for reproductive output and survival. These physiological costs, even in otherwise suitable habitats, call for careful consideration of regulating disturbance regimes. To reconcile habitat maintenance with species protection, conservation efforts should prioritize the management of the disturbances. This may include restricting vehicle access during breeding periods, designing alternative routes to avoid core breeding areas, and periodically maintaining ruts or pond‐like depressions outside critical activity periods of amphibians, using ethically appropriate ways.

## 6 Limitations

While detection probability was not explicitly modeled due to the absence of survey‐level covariates, our survey timing and protocol were designed to maximize detectability. Adult detection probability was high (*p* > 0.78), but lower for eggs and larvae (*p* = 0.57). The absence of formal modeling may introduce unquantified bias, particularly under conditions of reduced visibility or larval crypticity. We encourage future studies to incorporate detection‐related covariates to improve inference under variable field conditions (Schmidt et al. 2005; Hantzschmann 2020).

## Author Contributions

**Tibor Hartel:** conceptualization (lead), data curation (lead), formal analysis (lead), funding acquisition (lead), methodology (lead), project administration (lead), resources (lead), software (equal), supervision (equal), validation (equal), visualization (equal), writing – original draft (lead), writing – review and editing (equal). **Cristian Valeriu Maloș:** data curation (equal), formal analysis (equal), investigation (equal). **Eliana Sevianu:** investigation (equal), methodology (equal). **Ionuț Pascu:** data curation (equal), formal analysis (equal), investigation (equal), methodology (equal), software (equal), writing – original draft (supporting), writing – review and editing (supporting).

## Conflicts of Interest

The authors declare no conflicts of interest.

## Supporting information


Data S1.

Data S2.

## Data Availability

The datasets supporting the conclusions of this study have been provided as [Link], [Link] accompanying the manuscript. These files are available to reviewers during the peer‐review process. Upon publication, the data will be made publicly accessible in accordance with the journal's data archiving policies.
